# SPAR-4 and sustainable operations: A meta-analytic breakthrough

**DOI:** 10.12688/f1000research.160388.1

**Published:** 2025-01-30

**Authors:** Madhu Arora, Vasim Ahmad, Arpit Walia, Saurav Negi, Jatinderkumar R. Saini, Rupesh Kumar

**Affiliations:** 1 Symbiosis Institute of Computer Studies and Research, Symbiosis International (Deemed University), Pune, Maharashtra, India; 2 Uttaranchal Institute of Management, Uttaranchal University, Dehradun, Uttarakhand, 248007, India; 3 Department of Transportation, Logistics and Safety Management, Modern College of Business and Science (Affiliated with University of Missouri, St. Louis and Franklin University, USA), Muscat, Oman; 4 Jindal Global Business School (JGBS), OP Jindal Global University, Sonipat, Haryana, India

**Keywords:** Operations, Technology, Analytics, Sustainability, SPAR-4, Bibliometrics, Meta-analysis

## Abstract

**Background:**

In a deglobalized world, operations are a major competitive force for industrialized economies, especially with the inclusion of the latest technologies. This study aims to meticulously examine the literature on analytics, operations and sustainability through assessment of contributing publications, nations, authors, and keywords that can provide new perspectives for the research domain.

**Methods:**

The SPAR-4 (Smart Prediction and Reporting for Analysis and Research) model when integrated with bibliometric analysis helps follow a systematic approach to conducting research review along with meta-analysis to evaluate important articles, trends, and major contributors in these fields. This study therefore underlines the changing significance of sustainability in operational strategies, assesses the interconnectedness of research areas, and identifies emerging themes.

**Results:**

The findings highlight the increasing significance of analytics in tackling sustainability issues, providing industry and academia with information to match operational procedures with sustainable development objectives.

**Conclusions:**

By deepening grasp of the research landscape, this study supports strategic decision-making for sustained competitive operational excellence for the policy makers and strategic decision-makers.

## 1. Introduction

An increasing variety of techniques is being developed across numerous industries to help businesses become more effective. There is currently growing interest in utilizing data from diverse sources to support this goal. These efforts are commonly referred to as “analytics”. Although it has become a buzzword, little research has been conducted in this area, as it is still an emerging domain. Analytics were used to analyze conceptual and functional differences. According to Cooper (2012), analytics is defined as “Analytics is the process of developing actionable insights through problem definition and the application of statistical models and analysis against existing and/or simulated future data.” Analytics pays more emphasis on the possibility of real-world application than focusing only on reporting or theoretical explanation. The results of the analytics process could lead decision makers to take different actions based on their values and other considerations that were overlooked during the analysis, as many management reports are unable to offer actionable insights with a sufficient degree of clarity ( McAfee et al., 2012; Raut et al., 2019).

Hence, the study of analytics is essential in the present scenario, considering that analytics can bring about a major transformation not only in the way it functions but also in its outcomes. The ability of a firm to harvest upon analytics, evaluate it for business insights, and outperform its competitors could certainly help determine strategic advantage ( Arora et al. 2025) . Analytics can bring numerous benefits, some of which may include an increase in return on investment, enhanced productivity, competitiveness, enhanced performance measurement mechanisms, and surplus creation for customers ( Gordon & Perrey, 2015; McAfee et al., 2012; Nalchigar & Yu, 2017). At the strategic level, analytics can guide decisions related to network design, product design, strategic sourcing, and so on. At the operational level, this can result in enhanced visibility, flexibility, and integrated supply chains ( Papadopoulos et al., 2017).

Current national and international governments’ research priorities and concerns are based on the belief that the survival and well-being of the Earth’s ecosystem will be compromised if the world’s industrial economy does not act immediately. For this, quick action and rules with clear objectives and results are required. Thus, a sustainable development strategy is essential for business operations ( López-Torres et al., 2019). Sustainability is a crucial challenge ( Arora et al., 2024).

Proposing and developing more comprehensive tactics for sustainability and operations that incorporate continuing sustainability goals guaranteeing a foresight that encompasses all makers and consumers, along with social, economic, and environmental issues, is also crucial.

Organizations must function within the framework of sustainable development using Environmental, Social and Governance (ESG) practices ( Ballester Climent, 2022). A worldwide appeal to action to save the environment and guarantee sustainability and prosperity for all people, regardless of their background, gender, or condition, is embodied in the 17 SDGs (Sustainable Development Goals). Sustainable development emphasizes aspects such as raising awareness of environmental issues, promoting technological advancement, and enhancing operations ( Saba & Pretorius, 2024).

As analytics in the domain of operations management are still developing, the present study aims to provide insights into the various aspects in which research has been conducted. This study presents an overview of the field’s current status in terms of contributions from different authors, nations, and journals, suggests emerging areas, and inspires researchers to collaborate and further expand knowledge in the domain.

**Figure 1.**
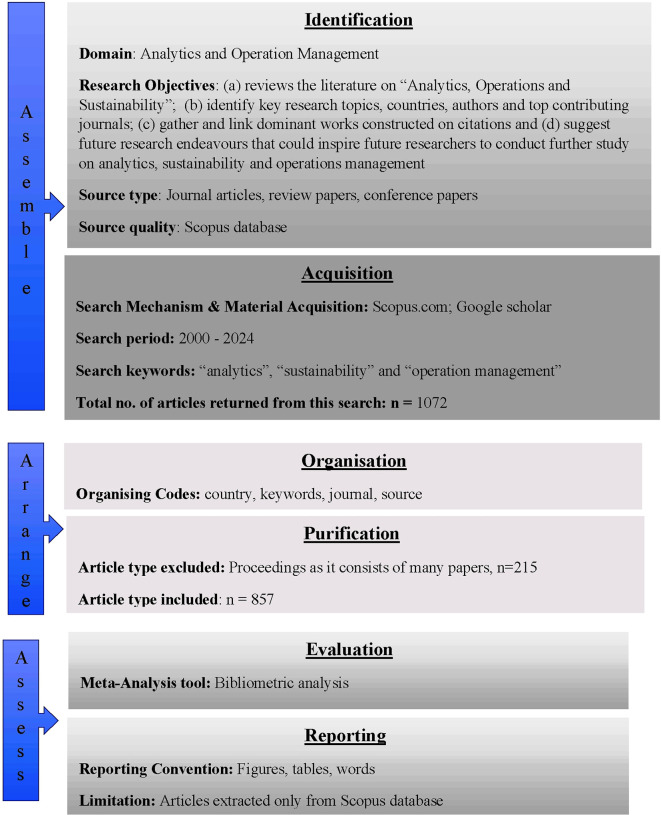
Literature review w.r.t SPAR-4 tool.

In addition to tracking the current and historical status of analytics and operation management research, this study is innovative in that it applies Paul’s et al. (2021) SPAR-4 (Scientific Procedures and Rationales for Systematic Literature Reviews) as shown in Figure 1, and creates a foundation for future research and application techniques. The major goal of this study is to evaluate the many different types of work that have been done in the domain and to perform analyses that will be helpful for further research in the field. Therefore, in order to address the analytics trends in the area of operations management, this study (a) reviews the literature on “Analytics, Operations and Sustainability” which starts from the year 2000; (b) offers an in-depth understanding of relevant domain by analysing 857 articles available since last twenty-five years using meta-analysis, and identifies key research topics, countries, authors and top contributing journals; (c) gathers and links the dominant works constructed on citations; and (d) suggests future research endeavors that could inspire future researchers to conduct further study on analytics, sustainability and operations management. The novelty of this study lies in the integrated approach that it applied by employing SPAR-4 and Meta-analysis achieved through bibliometric analysis as a tool to conduct an all-inclusive evaluation of literature using analytics, operations management, and sustainability as keywords.

A bibliometric review serves as a reliable guide for future studies and is a contemplative method for examining the past and identifying potential avenues for future investigations ( Ghosh, 2024). The researchers started with 1072 papers which were then screened to include 857 relevant articles. The study’s conclusion provides further information about the present status of the domain and highlights possible avenues for future investigation. The literature on analytics, operations management, and sustainability is reviewed in the next section, followed by the exploration methodology. Then, a systematic breakdown is presented using meta-analysis, and the paper concludes and imposes a research scope. Excel and VOSviewer software (an open source software) were used to analyse academic publications to find patterns and trends in the field of study ( Cavalcante, Coelho & Bairrada, 2021).

## 2. Review of literature

### 2.1 Analytics

In current dynamic times, it is difficult to ignore the revolution set by both sustainability and business analytics. Business analytics is fundamentally about using data to create value. Data is no longer referred to as the “sludge of the information age,” but rather as “the new oil” ( Acito & Khatri, 2014; Kumar et al., 2021a). Aligning strategy and desired behaviors with operations management, together with analytical activities and capabilities, is necessary to extract value from the data. According to a Boston Consulting Group (BCG) report on Industry 4.0, there are nine technologies that will be significant in bringing about transformation in the industrial environment: Data and Analytics, robotics, simulation, integration, IoT, cybersecurity, cloud, augmented reality, and additive manufacturing ( Rüßmann et al., 2015).

The ability of a company to extract data, analyze it for business insights, and gain an edge over its rivals could determine its competitive edge ( Oh et al., 2012; Wong, 2012). However, a clear operational requirement, fact-based decision-making culture, robust data infrastructure, necessary analytic tools, and qualified analytical staff within a suitable organizational structure are essential for business success ( Watson, 2013; Kumar et al., 2015; Bangwal et al., 2022b).

Based on the extent to which analytics is utilized in a business, it can be categorized into four types: (1) exploratory analytics, which detects relationships among parameters and analyzes them for insights and discovery; (2) descriptive analytics, which considers historical information in an easily comprehensible manner and arranges the acquired information and presents it as visual aids such as graphs, charts, maps, etc. for data implications; (3) predictive analytics, which extrapolates what is anticipated to occur in the future by using technologies such as machine learning, neural networks, and time series analysis; and (4) prescriptive analytics, which directs decision-makers towards accomplishing an objective through optimization of solutions to existing challenges ( Beer, 2018; Rajaraman, 2016; Delen & Demirkan, 2013; Bose, 2009).

Different kinds of capabilities—decision, analytical, and information—require a multitude of technologies ( Cao et al., 2019; Ferraris et al., 2019). The term ‘decision capabilities’ describes instruments, including dashboards and reports, that facilitate the distribution of information needed to make decisions ( Kowalczyk and Buxmann, 2014; Kumar & Kansara, 2018). These tools facilitate the communication necessary for decision making, in addition to the visual representation of insights ( Kumar et al., 2014; Wang and Byrd, 2017). A portfolio of tools and techniques for analysis is referred to as an analytical capability ( Sazu and Jahan, 2022). Descriptive, diagnostic, predictive, and prescriptive analytics are supported by these technologies, which also include traditional ad hoc queries, inferential statistics, predictive analytics, simulation, and optimization. Lastly, technologies that aid in the description, organization, integration, and sharing of data assets are referred to as information capabilities.

### 2.2 Analytics and Operations Management

In the last few years, research in operations management has observed a shift primarily owing to the expansion of data availability. Richer and more exhaustive data are becoming accessible ( Mišić and Perakis, 2020) in different types of industries, including manufacturing, healthcare, and retail. Hazen et al. (2018) also proposed that research in the field of operations can be instrumental in solving analytics problems in the domain of operations and supply chain management. There have been significant contributions in various domains directly or indirectly associated with data analytics and thus it becomes imperative to also focus on ‘data integration’ in operational decision-making ( Kumar et al., 2013; Feng & Shanthikumar, 2022; Gupta et al., 2024).

Considering the current business scenario to handle uncertainty in decision-making, firms must be adaptable and nimble. Operations have recently begun, including socially and ecologically responsible objectives, along with traditional performance objectives. Fortune 500 companies use green washing to leverage sustainable operations and strategies as a strategic advantage. Significant research is required to understand how social and environmental factors are integrated into various aspects of operational management ( Gunasekaran & Subramanian, 2018). Araz et al. (2020) also suggest analytics for operational risk management. Managers and organizations need to use operations management-based decision tools to solve data-driven problems for effective decision-making and thus highlight the interdependence between analytics and operations management ( Dutta et al., 2017; Kashav et al., 2022). It has also been argued that operational risk management is critical for any organization; not only are analytical tools evolving faster, but their effective application is crucial for the success of the organization ( Araz et al., 2020; Arora et al., 2023).

### 2.3 Sustainability and operations

Lack of resources to meet the demands of production presses the need for the advancement of sustainable operations ( Beltrán-Esteve and Picazo-Tadeo, 2017; Rajani et al., 2022). In the Brundtland Report (1987), the United Nations defined sustainable development as “meeting the needs of the present without compromising the ability of future generations to meet their own needs.” Therefore, every stakeholder must act responsibly to ensure the creation of policies and practices that support an economic model that prioritizes the production of environmentally clean, socially conscious, and financially successful goods ( Dey et al., 2011).

Operations and supply chains are vulnerable to hazards such as inadequate resource planning, unavailability of suppliers, underutilization of plants, and labor shortages, which can lead to delays and interruptions ( Vidal et al., 2024). Incorporating sustainability into operations will benefit not only manufacturers but also all stakeholders, resulting in reduced losses and wastage, enhanced performance, ultimately leading to enhanced profitability, coordination, and communication, and promoting the apportioning of resources as well as competencies across partners ( Kumar et al., 2012; Arora et al., 2023).

Some initiatives to incorporate business sustainability into operations include cleaner production ( Ikram et al., 2021; Zeng et al., 2010; Fijał, 2007), green lean, green lean six sigma, green manufacturing ( Kumar, 2020; Gholami et al., 2021; Garza-Reyes, 2015; Chugani et al., 2017), green supply chain management ( Tseng et al., 2019; Green, Zelbst, Meacham & Bhadauria, 2012) and circular economy ( Morseletto, 2020; Barreiro-Gen & Lozano, 2020; Geissdoerfer et al., 2017; Bangwal et al., 2022a). Stakeholders must now develop a greater understanding and innovativeness to comprehend and incorporate sustainability into their business operations.

Kosa and Dhliwayo (2024) in their study emphasized on the term “"digitalization of social innovation” which clearly highlights the incorporation of technology in operations for enhanced sustainability. When applied to operations, sustainability has significant benefits for performance, including reduced expenses, improved product quality and delivery, increased volume flexibility, and overall flexibility ( Magon et al., 2018). Green (2012), Liu and Huang (2012), and Yang (2010) emphasized the promotion of sustainability, as it also results in gaining competitive advantage. Collaboration between participants can enhance supply chain efficiency and contribute to environmental enhancement ( Huang and Yao, 2021).

## 3. Methods

### 3.1 Phase I - SLR (systematic literature review) using SPAR-4

Thomé et al. (2016) proposed a step-by-step approach to a systematic literature review. A systematic approach, while ensuring that no significant step is missed, also helps with easy comprehension and application. In this study, SPAR 4 ( Paul et al., 2021; Kumar et al., 2015) was applied as a six-step systematic literature review process to identify influential contributions, current research interests, and present recommendations for future research in the domain under study ( Figure 1).

According to the SPAR-4 tool, the SLR can be divided into three main stages:

**Phase I: Assemble** Two sub-stages comprising this stage as well: (i) Identification: The researcher chooses the field in which to carry out the investigation. Research questions were developed once the literature was reviewed. The types of articles to be extracted are also indicated. (ii) Acquisition: Understanding and application of the extraction period, search criteria, and extraction source. The researcher will extract the n’ number of publications after acquisition.

**Phase II: Arrange** There are two substages in Stage II: (i) Organization: The methodology for organizing the data analysis is described here. (ii) Purification: It is crucial to eliminate cases that do not add value to this study. This involves a purification procedure, following which the final count of the included articles will be decided.

**Phase III: Assess** There are two sub-stages inside this stage as well: (i) Evaluation: the agenda proposal process and the analysis of literature data are highlighted here. (ii) Reporting the analysis using tables, figures, and other formats. Restrictions and available sources of assistance are also noted.

### 3.2 Phase II - Meta-analysis using Bibliometrics

**3.2.1 Keywords**

Operations management and analytics were the two main keywords selected for data extraction and to ensure that the domain was well covered. The search string used for this purpose was (“operations management” OR “operation management”) AND ((“data analytics” OR “analytics”) OR (“sustainability” OR “sustainable operations” OR “ESG”)).

**3.2.2 Initial data gathering**

For the purpose of data acquisition, the Scopus database was considered, as it possesses a huge collection of data with numerous titles and is most popularly referred ( Yong-Hak, 2013; Kumar et al., 2014). The keywords were searched in “title, abstract, keywords” of the scopus database in Aug2024. The period considered for the search was 2000-2024. Only the English language was considered a popularly understood and read language. Initially 1072 articles were exported in the form. csv. However, after refining it there were 857 articles included in the final analysis. Books, book chapters, conference proceedings, unpublished articles, and magazine articles are excluded from the filtering process.

**3.2.3 Integration of SPAR-4 LR protocol and Bibliometric perspective**

The integration of SPAR-4 and bibliometric analysis is presented in Table 1, highlighting the stages, criteria, and related actions.

**Table 1.** SPAR-4 and Bibliometric integrated.

Stage	Criterion & related action	Rationale
**Assembling –** **(i)Identification**	**Domain – Analytics & Operation management with Sustainability as focus**	
	**Research Objectives** (a) review literature on “Analytics and Operations management along with sustainability” which dates back to 2000 (b) offer thorough understanding of the domain by analysing 857 articles published in past 25 years using the techniques of bibliometrics, and identifies key research topics, countries, authors and top contributing journals (c) gather and link the most dominant works constructed on citations; and (d) suggest current and developing research clusters that could inspire future researchers to conduct further study on analytics, sustainability and operations management	to direct the exploration of the domain's bibliometrics with respect to relationships and their linkages. To develop agenda for the domain.
	**Source types** Duplicate information or incomplete information, entries showing only one row for Conference proceedings containing many papers were excluded.	To include only relevant cases.
	**Source quality** Papers listed with Scopus database, were considered	Journals listed in Scopus are good quality journals.
**(ii)Acquisition**	**Search mechanism** Data was extracted from the export feature of Scopus database	To get good quality studies.
	**Search period** The period considered for extraction was 2000-2024	Though period considered included data from 2000 onwards however when data was extracted there were hardly any papers related to **analytics** prior to 2009.
	**Search keywords** TITLE-ABS-KEY (“operations management” OR “operation management”) AND ((“data analytics” OR “analytics”) OR (“sustainability” OR “sustainable operations” OR “ESG”)) was the search string on the basis of which data was extracted	To get studies relevant to the domain.
**Arranging –** **(i)Organisation**	**Organising Codes** Considering that Bibliometric analysis is to be performed the organising code includes source, journals, document type, keywords and country.	To get a broader understanding with reference to work done in different countries, etc.
**(ii)Purification**	**Article type excluded** The dataset was purged of entries that had duplicate information or incomplete information, conference proceedings with only 1 row but containing many papers. Out of a dataset of 1072, 215 articles were excluded as they were either not relevant to the domain under study or had incomplete information required for the study.	To get only relevant papers from peer-reviewed studies.
	**Article type included** After excluding 215 articles, remaining 857 articles were considered for further analysis.	
**Assessing –** **(i)Evaluation**	**Analysis method** Bibliometric analysis was performed on keywords, cluster, citations, publications, country, source, journals together with application of SPAR-4 tool for ease of understanding.	To understand the contribution of countries, authors, journals to the subject area.
	**Agenda proposal method** Gaps and areas for future research is identified.	Based on identification of gaps and future research areas, researchers and academicians can accordingly target upcoming research publications.
**(ii)Reporting**	**Reporting convention** Number of publications and citations have seen an increasing trend in the last two decades. 2012 onwards the number of publications and citations see a steady upward trend. ‘Production and operation management’ journal had highest number of citations followed by ‘Journal of cleaner production’. USA had maximum publications followed by China and India being at the fourth position while when citations are considered, again USA holds first position followed by UK and Brazil at the third. Words having Sustainability have highest occurrences and words with analytics have very few occurrences.	Helps summarize the key findings.
	**Limitation** The study is an analysis of the literature only and data is extracted only from Scopus database.	

## 4. Analysis and Results

### 4.1 Publication trend analysis

**Figure 2.**
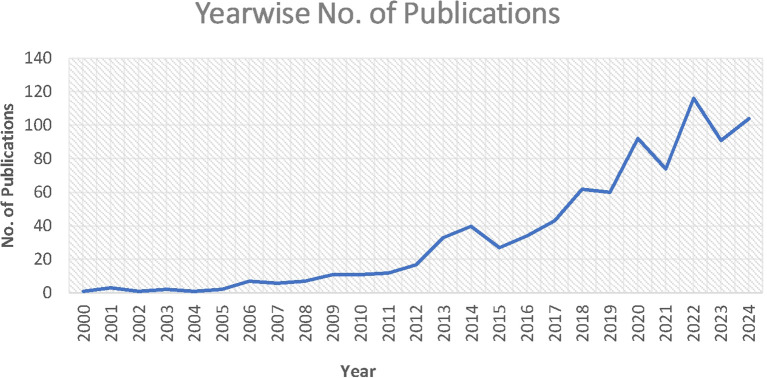
Year - wise number of publications.

**Figure 3.**
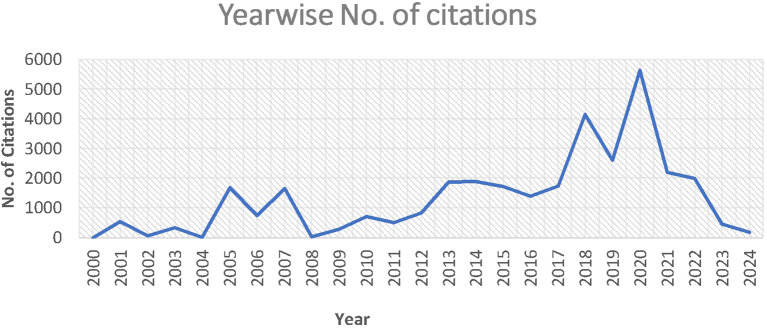
Yearwise number of citations.

The growth and progress of analytics and sustainability in operations management are demonstrated by the total number of publications and citations. A total of 857 cold chain papers gathered between 2000 and Aug 20, 2024 contained 33257 citations. The research fields’ publication and citation trends are displayed in Table 2 ( Figure 2 & Figure 3).

**Table 2.** Year - wise number of publications and citations.

Year	Publications	Citations	Year	Publications	Citations
2024	104	192	2011	12	510
2023	91	453	2010	11	725
2022	116	2003	2009	11	288
2021	74	2199	2008	7	16
2020	92	5636	2007	6	1656
2019	60	2615	2006	7	757
2018	62	4154	2005	2	1680
2017	43	1743	2004	1	6
2016	34	1403	2003	2	332
2015	27	1714	2002	1	52
2014	40	1880	2001	3	533
2013	33	1869	2000	1	1
2012	17	840	Total	857	33257

Figure 2 shows that the number of publications in the domain showed an increasing trend. Until 2012, the growth was not significant, but in 2012, there was a steady rise, and a steep rise further in 2020 and 2022. It is expected to increase further in the coming years with the growing utility of analytics and environmental concerns.

Figure 3 clearly shows a sturdy upsurge in citations, reflecting a steep increase in 2017 and 2019. This is an indication of the increasing interest of researchers in the area of analytics and sustainability, as applicable in the area of operation management. Therefore, the domain under study is gaining scholarly attention and influence, which is indicative of the expanding significance of analytics and sustainability in the operational management domain.

As shown in Table 3, ‘Production and Operations Management’ and ‘Journal of Cleaner Production’ have the highest citation counts, reflecting their significance in this domain.

**Table 3.** Top 5 Journals on the basis of citations.

Journal	Citation	Publications	Publisher	Citescore	Impact Factor	SNIP	h-index
Production and Operations Management	5533	51	Wiley Blackwell/Sage	7.5	4.8	1.723	138
Journal of Cleaner Production	3735	34	Elsevier	20.4	9.7	2.236	309
International journal of operations and production management	1960	25	Emerald	13.3	7.10	2.08	163
International Journal of Production Research	1713	26	Taylor & Francis Ltd.	19.2	7.0	2.724	186
Journal of Operations Management	1615	9	John Wiley & Sons	11	7.8	2.46	219
Annals of Operations Research	1322	18	Springer Netherlands	7.9	4.4	1.396	125
International Journal of Production Economics	1193	17	Elsevier B.V	21.4	9.8	2.88	231
Manufacturing and Service Operations Management	1107	19	INFORMS Institute for Operations Research and the Management Sciences	9.5	4.8	3.27	110
Sustainability (Switzerland)	1084	54	MDPI	5.8	3.3	1.31	169
Management Science	1036	8	INFORMS Institute for Operations Research and the Management Sciences	7.7	4.6	3.19	290

As indicated by the table above, although there are few publications, the increasing number of citations reflects growing interest in the area.

The United States leads in citations for research on analytics and operations management, as illustrated in Figure 6 Other notable contributors include the UK and Brazil, reflecting global research trends.

**Figure 4.**
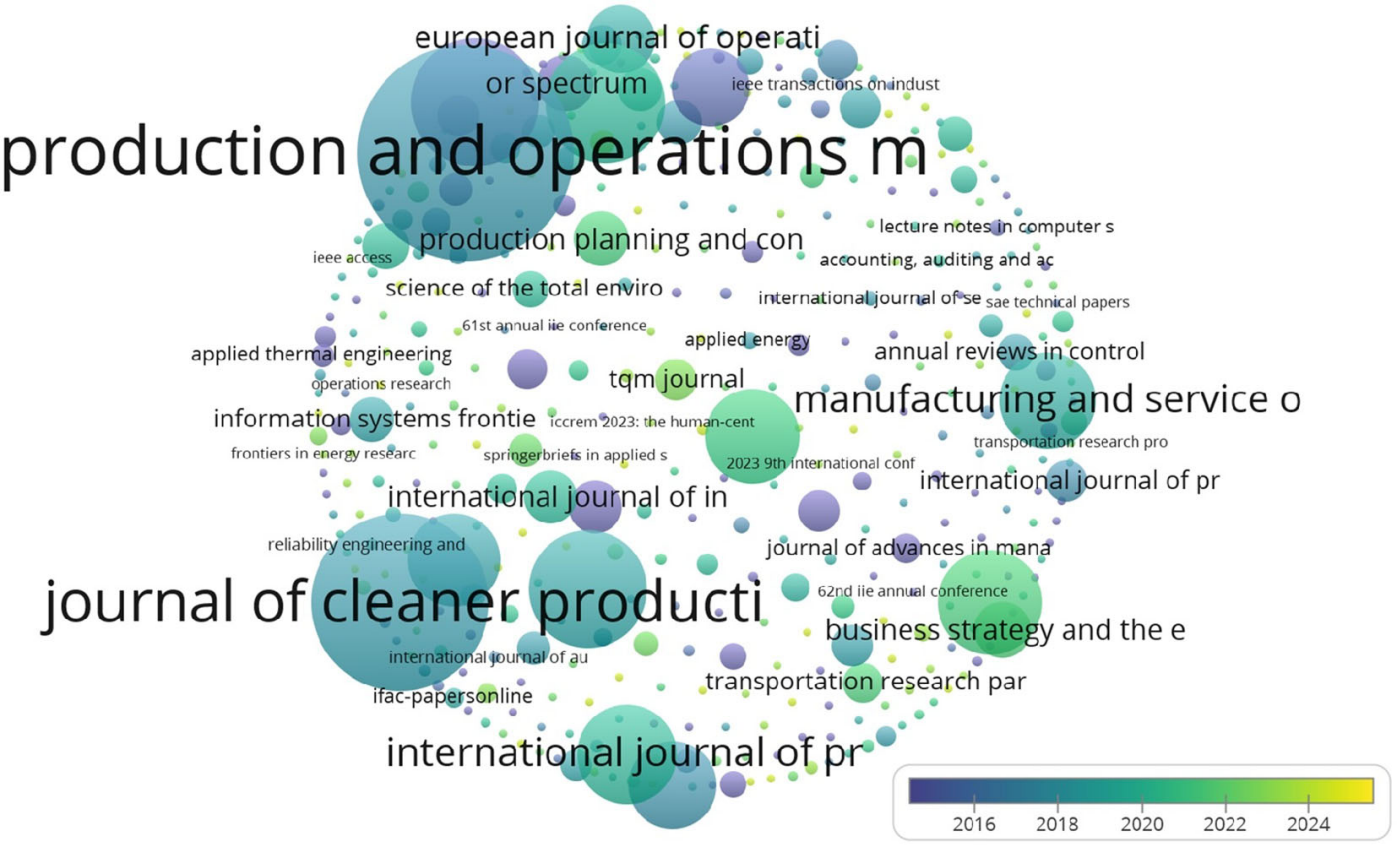
Source map (citations) [Overlay visualization].

**Figure 5.**
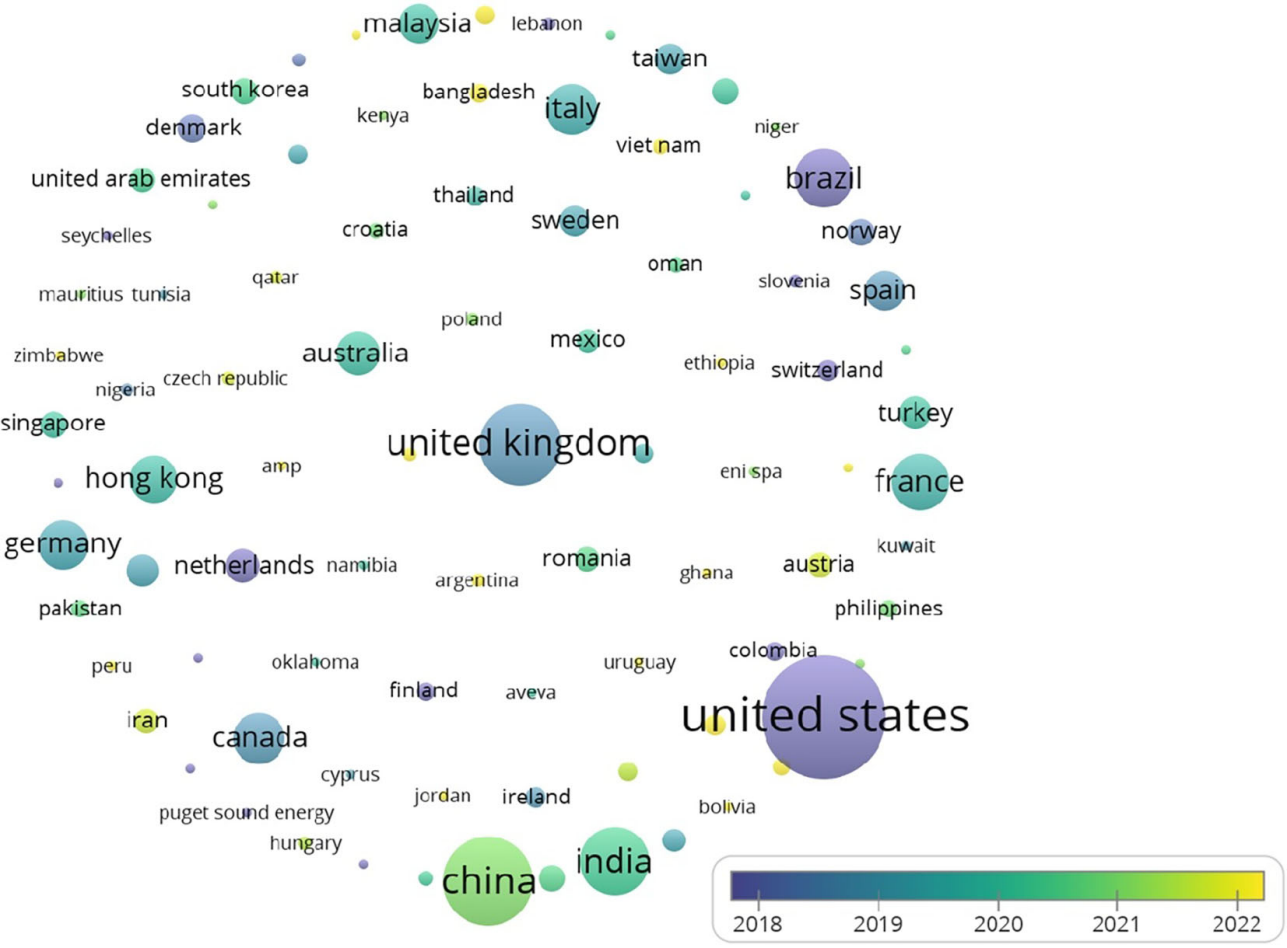
Contribution of top 5 countries (based on publications).

As indicated by Table 4 and Figures 4 and 5, the USA holds the first rank in both the number of publications and citations. While India is at 4th place where the number of publications is considered, there are few citations (2634). One can interpret that compared to the number of publications, the number of citations is significant. This reflects the pressing need to conduct more research in the area, as not many studies have been conducted; however, there is a growing interest as well as the need for further investigation in the realm of analytics in operations.

**Table 4.** Contribution of countries (publications, citations).

Country	No. of publications	Country	No. of citations
USA	250	USA	11354
China	133	UK	6621
UK	113	Brazil	4224
India	76	France	4647
Brazil	59	China	3389

**Figure 6.**
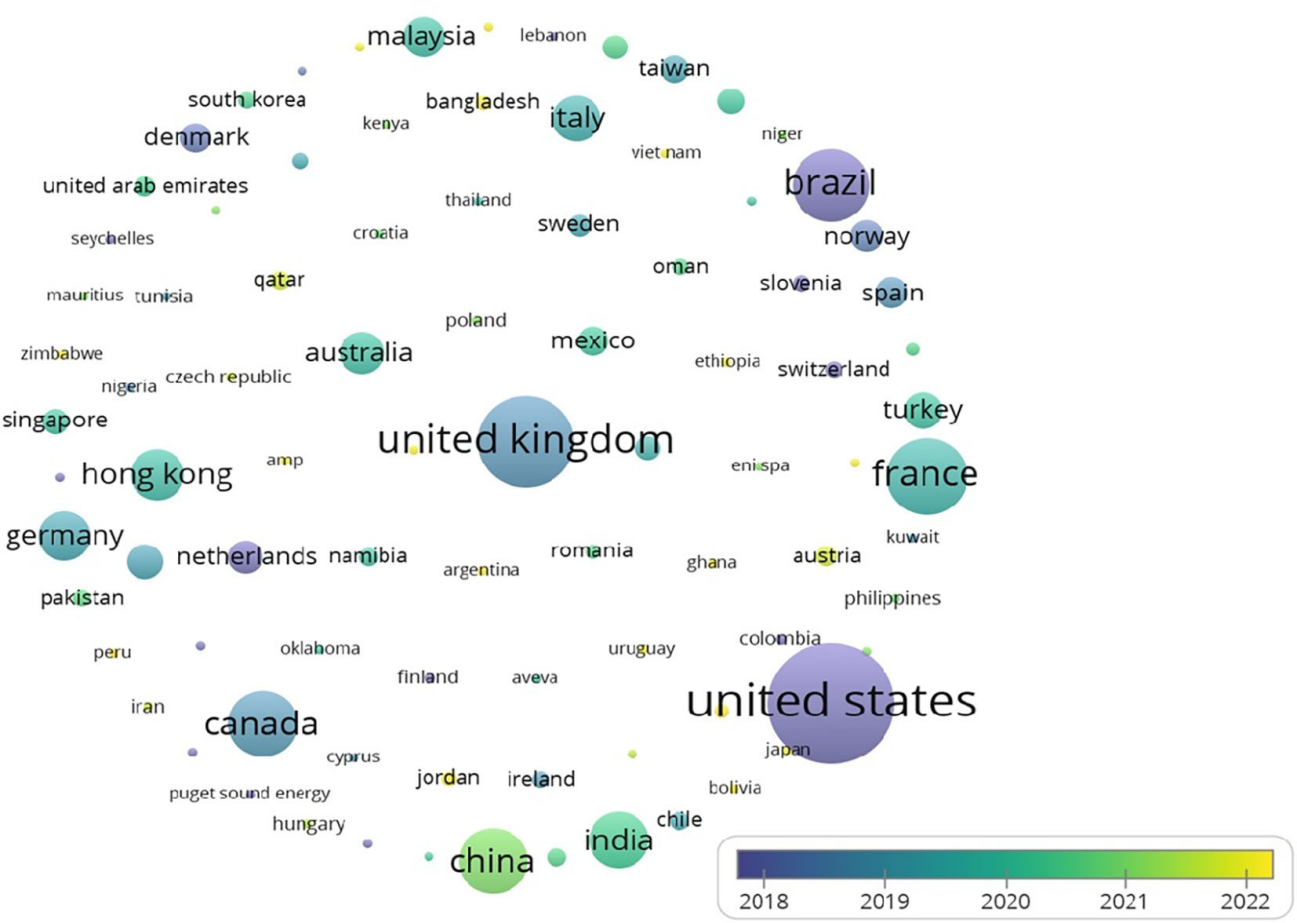
Contribution of top 5 countries (based on citations).

**Figure 7.**
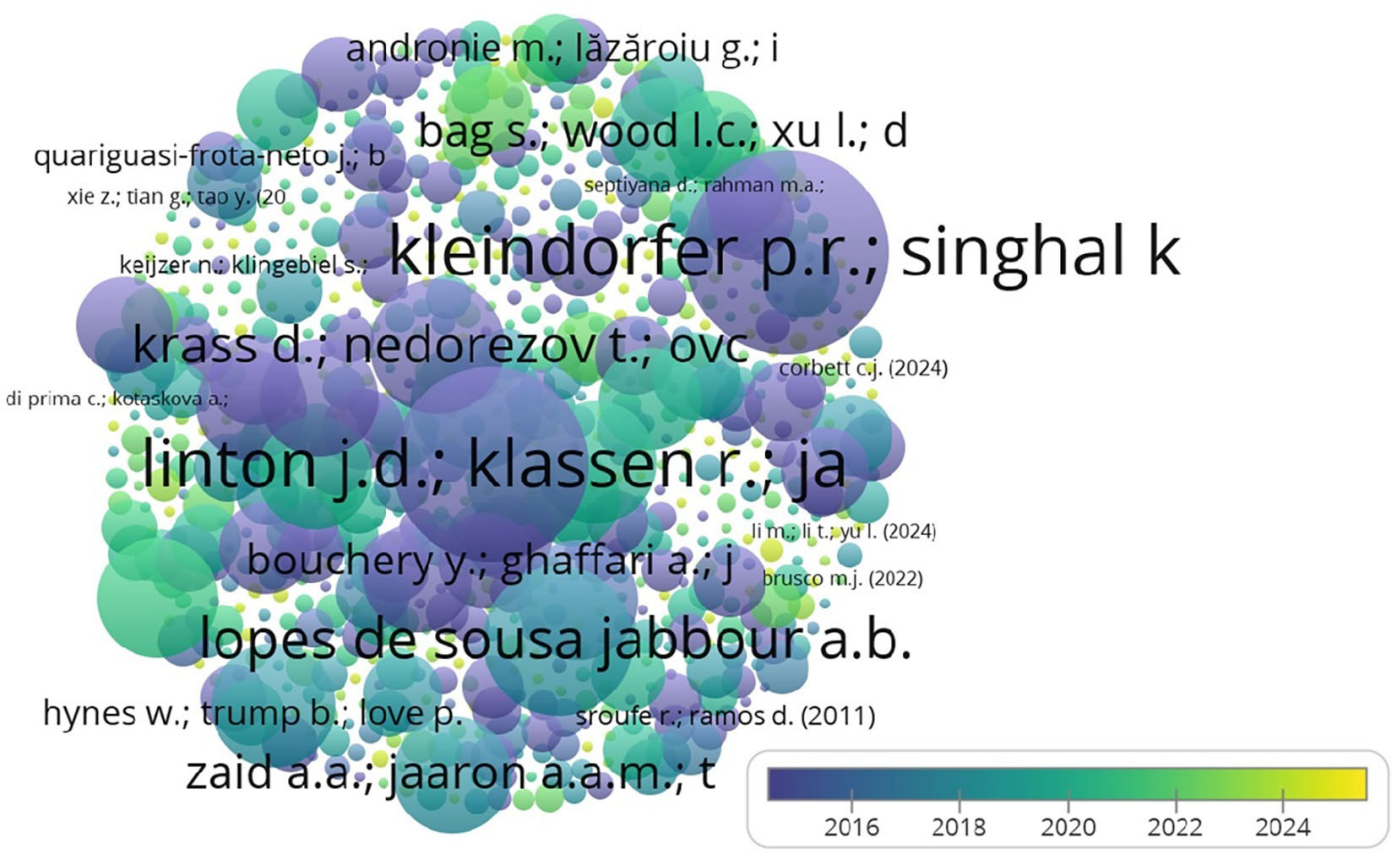
Top 5 contributing authors (citations).

The top contributing authors in the domain are presented in Figure 7, with Kleindorfer et al. making the most significant impact in terms of citations.

Table 5 highlights the most cited authors, with Kleindorfer et al. leading the field in sustainable operations management.

**Table 5.** Top 5 contributing authors (citations).

Authors	Title	Source	Year	No. of Citations
Kleindorfer, Singhal, Van Wassenhove	Sustainable Operations Management	Production and Operations Management	2005	1369
Linton, Klassen, Jayaraman	Journal of Operations Management	Sustainable Supply Chains: An Introduction	2007	1266
Loupes de Sousa Jabbour, Jabbour, Godinho Filho, Roubaud	Annals of Operation Research	Industry 4.0 and the Circular Economy: A proposed research agenda and original roadmap for sustainable operations	2018	756
Krass, Nedorezov, Ovchinnikov	Production and Operations Management	Environmental taxes and the choice of green technology	2013	602
Nascimento, Alencastro, Quelhas, Caiado, Garza-reyes, Lona, Tortorella	Journal of Manufacturing technology management	Exploring industry 4.0 technologies to enable circular economy practices in a manufacturing context: a business model proposal	2019	587

**Figure 8.**
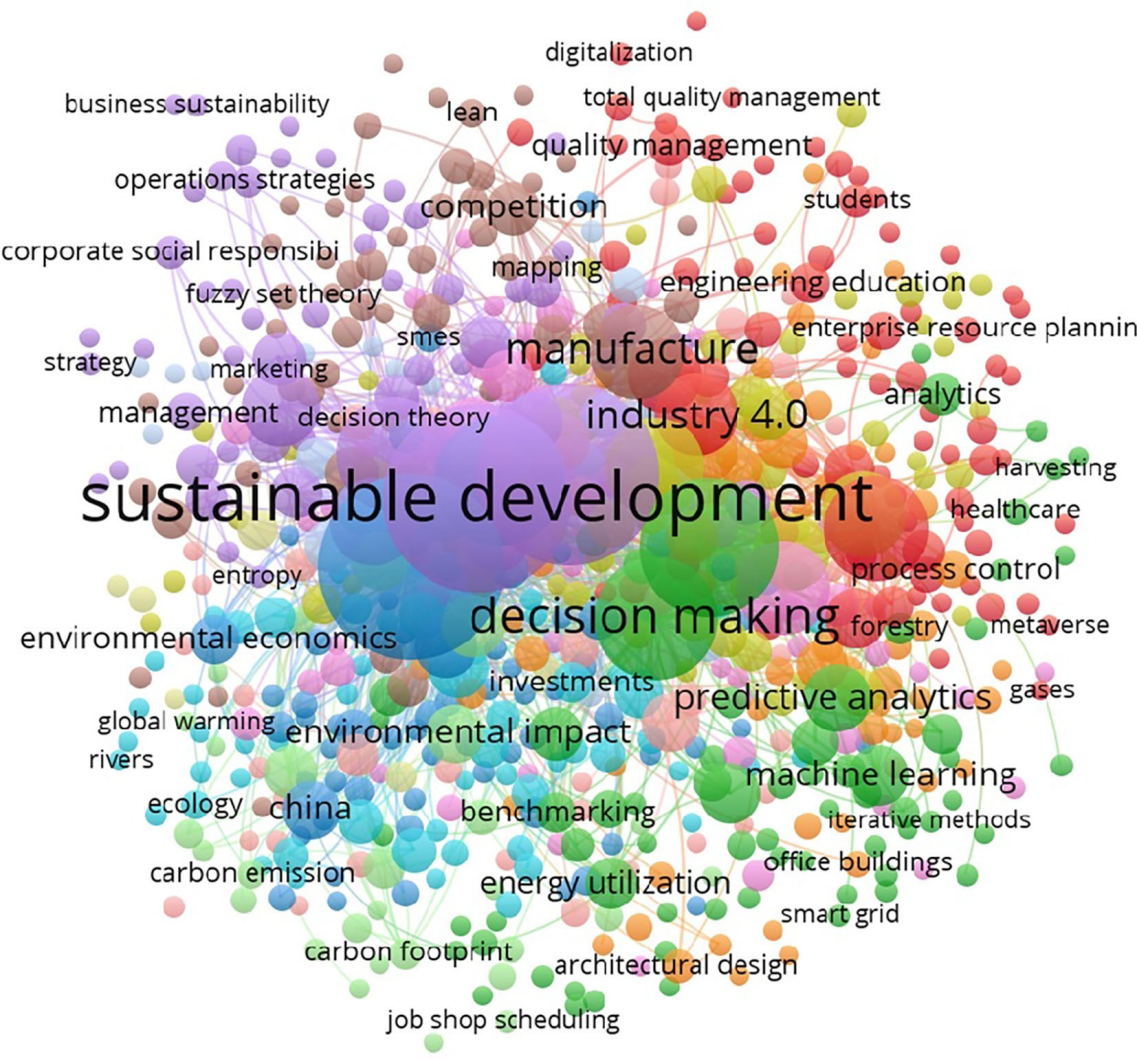
Keyword Co-occurrences (All keywords).

Figure 8 depicts the co-occurrence of keywords in the reviewed literature, with ‘sustainability’ and ‘operations management’ emerging as the most connected terms.

**Figure 9.**
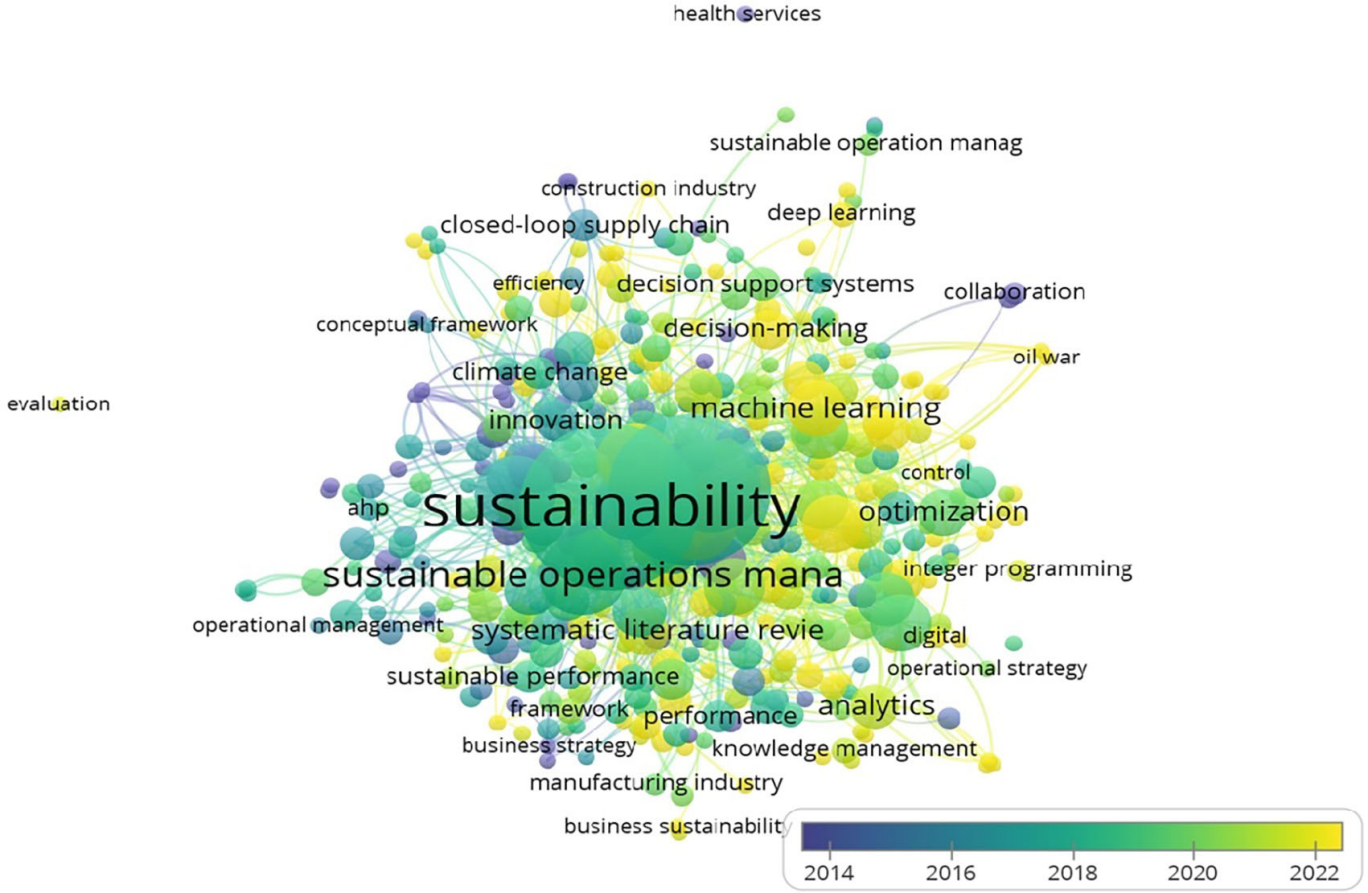
Keyword co-occurrences (author keywords).

The network of author-specific keywords is shown in Figure 9, revealing the prominence of terms such as ‘big data,’ ‘machine learning,’ and ‘supply chain’ in recent research.

The most frequently used keywords, such as ‘sustainability’ and ‘operations management,’ are summarized in Table 6.

**Table 6.** Top contributing keywords.

Keyword	Occurrences	Link strength
Sustainability	180	519
Operations management	146	410
Sustainable operations management	42	106
Sustainable operations	37	96
Artificial Intelligence	21	73
Big Data	21	56
Manufacturing	21	67
Machine learning	20	47
Supply chain	19	51
Data analytics	19	46
Analytics	13	36

**Figure 10.**
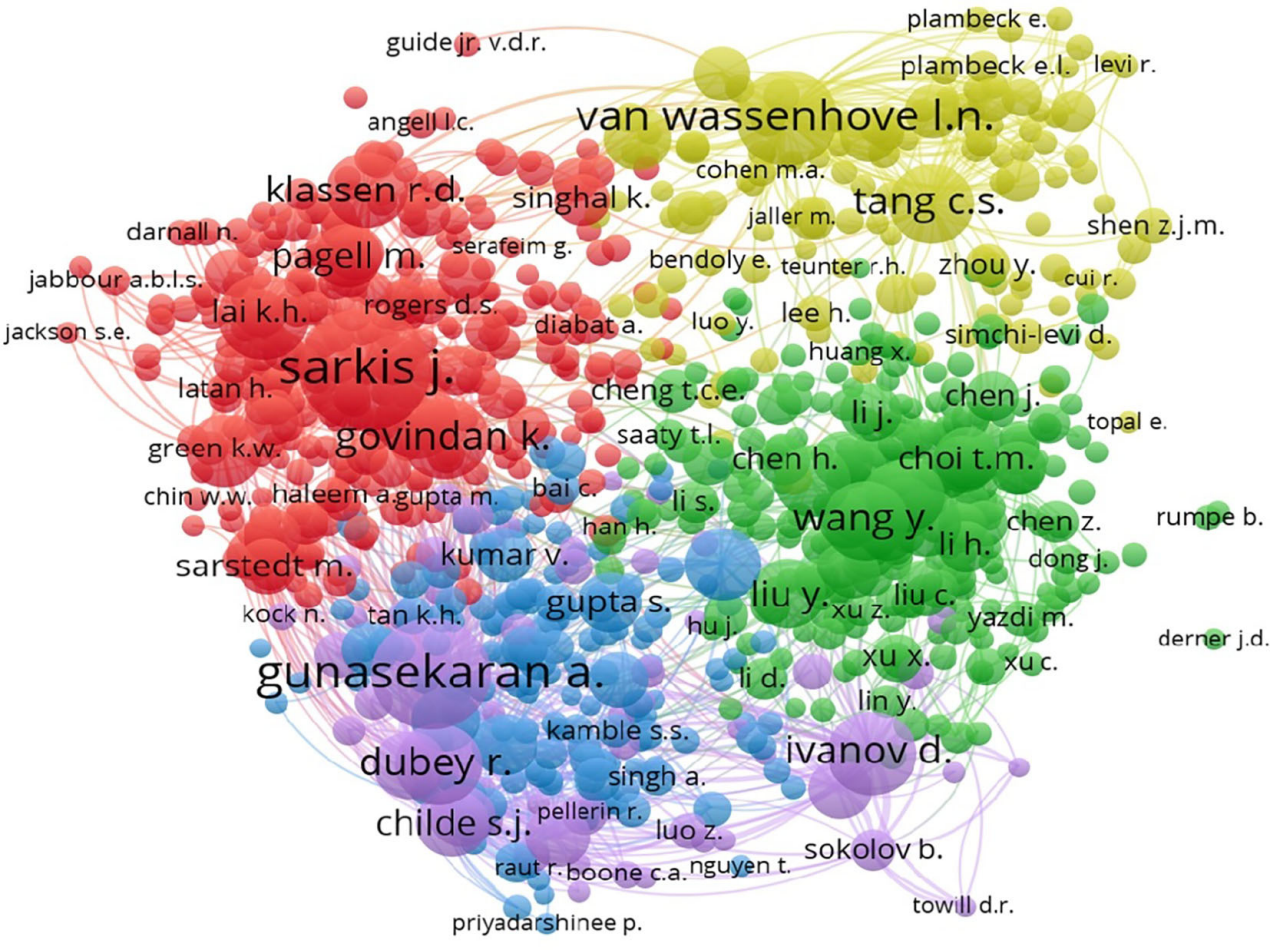
Co-citation authors network.

The co-citation network illustrated in Figure 10 highlights the influential authors in the field, with Sarkis J. and Gunasekaran A. showing strong connections.

The co-citation analysis, as detailed in Table 7, reveals Sarkis J. as the most frequently cited author, indicating his influence in this field.

**Table 7.** Top co-cited authors.

Author	Citations	Link strength
Sarkis J.	496	37730
Gunasekaran A.	418	41449
Van Wassenhove L.N.	307	22080
Zhu Q.	245	18662
Ivanov D.	228	20577

## 5. Discussion

The findings of the bibliometric analysis demonstrate how analytics and operation management research is evolving. It assists scholars in identifying the leading nations, works, and writers that influence the research, in addition to the primary research themes, trends, and gaps in the body of knowledge. According to this study, the United States, China, the United Kingdom, and France are the top four nations driving research on sustainability, operation management, and analytics. These nations have strong ties in collaborative research projects and a comparatively high number of publications. Additionally, the top three journals — Production and Operations Management, Journal of Cleaner Production, and International Journal of Operations and Production Management — have become crucial forums for exchanging research in these fields. Significant contributions have been made by Kleindorfer et al. The fact that their work received high citations indicates how much impact they had on the direction of the discussion.

Research gaps were found in studies concerning the use of analytics in sustainable operations and their impact on sustainability. These knowledge gaps point to the need for more research to deepen our understanding of analytics for sustainable operations that promote growth. The findings of the bibliometric analysis offer insightful information on global trends in analytics and operational management research. It displays the top nations, works, writers, primary research themes, trends, and gaps in the literature. As they set the foundation for further research and support the ongoing evolution of these professions, the conclusions of the review are significant for experts and scholars.

Worldwide research trends revealed the most significant lessons in Analytics and Operation management with a focus on sustainability through meta-analysis utilizing bibliometrics as a tool, including:

1. Contributing nations, publications, and authors: USA, China, UK, and India were found to have prominent contributions in the analytics and operation management domain based on the number of publications, while USA, France, UK, and China were realized as significantly contributing in terms of citations. Furthermore, important writers such as Gunasekaran, Ivanov, Akter, and Wamba have made important contributions in terms of citations received for their works.
2. Main areas of study: The main areas of study were the use of technologies in operation management and the significance of analytics in operation management. These themes show that people are becoming more aware of how analytics and technology can help make operations more productive and effective, and help gain competitiveness. Sustainability remains the focus, considering present and futuristic concerns.
3. Patterns and Gaps in the Literature: The study found that there are gaps in the literature, especially when it comes to looking into new trends, such as the role of sustainability in the use of analytics in operation management, the effect of digital transformation on operation management, and how analytics is being used in operation management. These gaps in knowledge mean that more research can be conducted in the future to learn more about these topics and help the fields of Analytics and Operation management grow.
4. Intellectual Collaboration and impact: This study revealed the manner in which prominent journals and researchers demonstrate worldwide interest in and cooperation to advance knowledge in the areas of Analytics, Sustainability and Operation management.

Based on the exhaustive literature review conducted coupled with the meta-analysis performed, it can be suggested that firms need to incorporate extensive use of analytics to enhance operational efficiency and improve their competitive quotient. It is vital that the function of operation management adopts the norms of sustainable management, as it profoundly influences many sustainability consequences ( Bettley & Burnley, 2008). Sustainability is imperative for firms to adopt and integrate in their operations ( Kumar et al., 2010; López-Torres et al., 2019; Magon et al., 2018; Longoni & Cagliano, 2015). Organizations that focus on sustainability tend to enhance their brand image in the eyes of their consumers and investors. Forecasting, inventory management, marketing, revenue management, supply chain management, transportation management, and risk analysis are some of the operational management areas where analytics can be used ( Kraus, Feuerriegel & Oztekin, 2020; Araz et al., 2020; Choi et al., 2018; Hazen et al., 2018). Employing Analytics will ensure better predictability, effective decision-making, and higher responsiveness.

### 5.1 Implications for Policy makers, decision-makers and strategists

Businesses must implement a framework of strategic flexibility to enable them to compete in the current business environment and prepare for a contentious global arena. To deal with these uncertainties, organizations should embrace flexibility and utilize the benefits offered by analytics to predict future outcomes, thereby reducing the extent of uncertainty. The authors therefore recommend that businesses capitalize on the advantages offered by analytics and integrate them into their operations. Analytics can significantly improve visibility, transparency, and performance in operations and supply chains ( Conboy et al., 2020; Zhu et al., 2018; Papadopoulos et al., 2017; Kumar et al., 2021b). Hence, firms can enhance their operational management-based sustainable business performance. Therefore, it is suggested that firms formulate policies and procedures for the enhanced integration of analytics and sustainability practices into their operations.

## 6. Conclusion

Much has been learned about the evolution and interconnections of the domains of operational management and analytics from the bibliometric examination of global research trends in these areas. According to the report, academic interest in integrating analytics into operation management models is currently very high. This demonstrates how the corporate sector is beginning to acknowledge the need for data and information ( Delen & Demirkan, 2013; Karmarkar & Apte, 2007; Dias, 2001). Additionally, conventional operation models are gradually being replaced by those that incorporate optimization and competitiveness requirements with increased emphasis on sustainability ( Söderholm, 2020; Jaehn, 2016; Eskandarpour et al., 2015; Tang & Zhou, 2012).

This demonstrates how current studies are beginning to integrate analytics into the management of operations. It also emphasized how crucial technological advancements are to the sustainability of corporate practices, particularly in the areas of digital and financial technologies. The results of this study provide a complete picture of the state of research in these fast-developing fields, as well as its potential future directions. Researchers can contribute to the ongoing development of the domains of analytics and operational management by investigating these prospective avenues and conducting empirical analyses to support this trend.

This study contributes to enhancing the understanding of operating a sustainable firm in the digital era and developing practical solutions for the global economy. For instance, emerging topics such as the impact of analytics on sustainable operations, the repercussions of digital transformation on corporate sustainability, and the maintenance of current news and its implications for sustainable company operations can be explored and examined further. Exploring the policy impacts of integrating analytics concepts into business models and operational practices is a potential future endeavor. Therefore, the results of systematic literature review and meta-analysis are valuable for academics and professionals, as they illustrate the current shape of exploration and could aid direct future investigation in the spheres of Analytics, Sustainability and Operation management.

## Data Availability

The data underlying this systematic review, including the PRISMA checklist, PRISMA flow diagram, and dataset obtained from Scopus, are publicly available on Figshare. The repository details are as follows: Repository: Figshare Project Title: Systematic Review on Analytics, Sustainability, and Operations Management DOI: https://doi.org/10.6084/m9.figshare.28152632.v1 ( Ahmad, 2025). Data are available under the terms of Creative Commons Zero (CC0) license. This data should be cited as: Arora, M., Ahmad, V., Walia, A., Negi, S., Saini, J.R., & Kumar, R. (2025). Systematic Review on Analytics, Sustainability, and Operations Management. Figshare. 10.6084/m9.figshare.28152632. The PRISMA checklist and flow diagram were prepared in accordance with the PRISMA guidelines and are included in the repository as extended data. This systematic review was conducted in accordance with the PRISMA guidelines. The PRISMA checklist and flow diagram have been completed and uploaded to Figshare as extended data, accessible at https://doi.org/10.6084/m9.figshare.28152632.v1 ( Ahmad, 2025).
