# Supplementary material for: SPAR-4 and sustainable operations: A meta-analytic breakthrough
Source: F1000Res. 2025 Sep 3;14:145. Originally published 2025 Jan 30. [Version 2] doi: 10.12688/f1000research.160388.2 (PMC12438951; doi:10.12688/f1000research.160388.2)
Supplement: Peer review history

# Reviewer report: Reviewer response for version 2

**DOI:** 10.5256/f1000research.186596.r413081

**Published:** 2025-09-15

**Authors:** Rakibul Hasan Chowdhury

**Affiliations:** 1 University of Portsmouth, Portsmouth, Portsmouth, UK

Summary of the Article

 The manuscript presents a **systematic literature review and bibliometric meta-analysis** of research at the intersection of analytics, operations management, and sustainability from 2000 to 2024. Using the SPAR-4 protocol and bibliometric techniques (VOSviewer, Excel), the authors identified 857 relevant publications from an initial pool of 1,072 retrieved through Scopus. The study highlights global publication trends, leading contributing countries, journals, and authors, while also analyzing keyword co-occurrence, co-citation networks, and thematic research clusters. The paper positions itself as an integrated contribution that combines structured review methodology with bibliometric mapping, aiming to guide future research, policymaking, and managerial practice in sustainable operations.

 Evaluation and Comments

 1. Title and Abstract

- **Title:** The current title is clear and descriptive, but somewhat generic. Consider a more precise alternative that emphasizes both the **systematic review methodology and bibliometric integration**, e.g., *“Analytics, Sustainability, and Operations Management: A Systematic Literature Review and Bibliometric Meta-Analysis Using SPAR-4”*.
- **Abstract:** The abstract is comprehensive, but slightly repetitive in describing the methodology. A more concise version highlighting (i) scope, (ii) methods, (iii) key findings, and (iv) main contribution would improve readability.

2. Scientific Soundness

- The manuscript is **scientifically correct overall**. The SPAR-4 framework is appropriate, and bibliometric analysis is a standard, recognized approach.
- However, several methodological clarifications are required to strengthen transparency and reproducibility (see below). These are **essential to make the paper scientifically sound**.

3. Methodology

- **Database limitation:** The study relies exclusively on **Scopus**. While Scopus is a strong source, the authors should justify excluding other major databases (e.g., Web of Science, IEEE Xplore). At minimum, they should discuss possible **coverage bias**.
- **Search string and filters:** The search protocol must be reported **exactly as executed** (Boolean strings, fields searched, filters, and date of extraction). This is crucial for replicability.
- **Screening process:** The flow diagram is referenced but not fully detailed. Authors should clarify (i) how duplicates were removed, (ii) who conducted screening (single vs. multiple reviewers), and (iii) what specific inclusion/exclusion criteria were applied.
- **Quality assessment:** While bibliometric studies often omit quality appraisal, it would strengthen the paper to include a discussion of study quality or acknowledge this limitation explicitly.

4. Results and Analysis

- The results are well-structured, with figures and tables providing useful insights. However:
  - Some figures are referenced (e.g., PRISMA diagram, author networks) but not shown in the manuscript. These must be included to ensure completeness.
  - The interpretation of co-citation and keyword networks could be deepened. Rather than only listing frequent keywords, the authors should explain the **emerging thematic areas** these clusters represent.
  - The statistical validation (t-test) is interesting but needs clearer explanation: What exactly is being compared pre- and post-2010, and why is 2010 chosen as the cut-off?

5. References

- The references are **sufficient and mostly recent**, with many from 2018–2024.
- To further strengthen the manuscript, the authors may consider including:
  - Floridi & Cowls (2021) on AI and sustainability principles.
  - Taddeo & Floridi (2021) on AI regulation in cybersecurity/operations.
  - More works from *Computers & Security* and *IEEE Transactions on Engineering Management* to broaden empirical grounding.

6. Ethical Issues

- No ethical issues are present, as the study relies on secondary data and bibliometric analysis. The authors should include a statement confirming that no ethical approval was required.

7. Competing Interests

- No competing interest issues are apparent. Authors should add a declaration: *“The authors declare no competing interests.”*

Required Revisions (Must Address for Scientific Soundness)

1. Provide the **exact search string, filters, and date of extraction** for reproducibility.
2. Clarify the **screening process**: duplicates, inclusion/exclusion criteria, and reviewer agreement.
3. Justify the use of **Scopus only or** discuss database coverage limitations.
4. Ensure all **figures and tables referenced in the text** (e.g., PRISMA, network maps) are included in the final manuscript.
5. Expand the **interpretation of bibliometric networks** (keywords, co-citations) to identify meaningful research themes.
6. Add a **formal declaration of ethics and competing interests**.

General Comments (Optional Enhancements)

- Shorten and tighten the abstract to avoid repetition.
- Consider refining the title to highlight the dual contribution (SLR + bibliometrics).
- Expand discussion of **global South research contributions** (e.g., Africa, Latin America) which are currently underrepresented.
- Provide practical implications in more detail: How can managers and policymakers use the insights beyond academia?

Recommendation: The manuscript makes a **relevant and timely contribution** to the literature on analytics, sustainability, and operations management. It is scientifically correct but requires **major revision** to improve methodological transparency, completeness of figures, and interpretative depth. Once these revisions are addressed, the article will be suitable for indexing.

Is the work clearly and accurately presented and does it cite the current literature?

Yes

If applicable, is the statistical analysis and its interpretation appropriate?

Yes

Are all the source data underlying the results available to ensure full reproducibility?

Yes

Is the study design appropriate and is the work technically sound?

Yes

Are the conclusions drawn adequately supported by the results?

Yes

Are sufficient details of methods and analysis provided to allow replication by others?

Yes

Reviewer Expertise:

Artificial Intelligence (AI) in Operations Management, Sustainable Operations and Supply Chains, Business Analytics and Decision Support, Digital Transformation and Industry 4.0, Data Privacy, Ethics, and Governance, Bibliometric and Systematic Literature Reviews, Environmental, Social, and Governance (ESG) Practices, Technology-Enabled Sustainability Strategies

I confirm that I have read this submission and believe that I have an appropriate level of expertise to confirm that it is of an acceptable scientific standard, however I have significant reservations, as outlined above.

# Reviewer report: Reviewer response for version 1

**DOI:** 10.5256/f1000research.176279.r402318

**Published:** 2025-08-11

**Authors:** Anurag Singh

**Affiliations:** 1 Birla Institute of Technology & Science Pilani - Dubai Campus, Dubai, Dubai, United Arab Emirates

The manuscript titled "SPAR-4 and Sustainable Operations: A Meta-Analytic Breakthrough" addresses a timely and important topic, offering valuable insights into the integration of SPAR-4 within sustainable operations frameworks. The meta-analytic approach is rigorous, and the findings contribute meaningfully to both theory and practice. Overall, the paper is well-structured and demonstrates strong potential for indexing. However, I recommend minor revisions to further enhance clarity, coherence, and alignment with the journal’s standards.

 Specific Comments:

 1.    Clarify certain statements – Some sentences, particularly in the results and discussion sections, could be rephrased for better clarity and ease of comprehension.

 2.    Strengthen referencing – Certain claims, especially in the literature review, would be more compelling if supported by recent and highly relevant citations from the last 3–5 years. Please see the following papers for your reference for improving the method and supporting your arguments:

 •    Does mindfulness moderate between incivility, aggression, and conflict at work? Findings from symmetric and asymmetric modeling approaches. 

 •    How does social media use enhance employee’s well-being and advocacy behaviour? Findings from PLS-SEM and fsQCA. 

 •    "If you want peace, avoid interpersonal conflict”: a moderating role of organisational climate

 •    An ounce of prevention is worth a pound of cure: A study on emotional regulation, thwarted social needs, disposable income and its relationship with psychological well-being

 •    Impact of COVID-19 on monitoring human health behaviour through digital medium systems. 

 3.    Check formatting consistency – Ensure uniform formatting of headings, tables, figures, and references in line with the journal’s style guide.

 4.    Give authors source for diagrams

 All the best for addressing the comment.

Is the work clearly and accurately presented and does it cite the current literature?

Yes

If applicable, is the statistical analysis and its interpretation appropriate?

Yes

Are all the source data underlying the results available to ensure full reproducibility?

Yes

Is the study design appropriate and is the work technically sound?

Yes

Are the conclusions drawn adequately supported by the results?

Yes

Are sufficient details of methods and analysis provided to allow replication by others?

Yes

Reviewer Expertise:

Management; Statistics; AI

I confirm that I have read this submission and believe that I have an appropriate level of expertise to confirm that it is of an acceptable scientific standard.

# Reviewer report: Reviewer response for version 1

**DOI:** 10.5256/f1000research.176279.r400349

**Published:** 2025-08-01

**Authors:** Md Rokibul Hasan

**Affiliations:** 1 Gannon University, Erie, Pennsylvania, USA

The paper provides a systematic literature review and meta-analysis of a study on the combination of analytics, operations management, and sustainability with the help of the SPAR-4 framework and bibliometric analysis. In the research, 857 peer-reviewed articles between 2000 and 2024 were retrieved and analyzed based on the Scopus database. The major contributions to the field are to name the most successful countries, authors, journals, and seminal pieces in this new area of interdisciplinary inquiry. This paper describes the development of the research trends, addresses the gap in research regarding the sustainability in operational analytics, and suggests the directions of future research. The outcomes indicate the increasing importance of incorporating analytics of sustainable operational excellence and strategic decisions. A transparency of the data is also present when the authors give their dataset and PRISMA checklist. The following are constructive criticisms and suggestions the authors should consider addressing:

1. The article manuscript is structured and has a logical sequence, especially in the application of the SPAR-4 model. It has extensive coverage of recent and relevant literature, especially of the high-impact journals. However, clarity is compromised by more than a bit of jargon, repetition (in the introduction and methodology in particular), and a few grammatical inconsistencies. Other sources are obsolete, un-peer-reviewed datasets (e.g., Figshare) and can be low on academic rigor.
2. The manuscript article largely uses bibliometric analysis (descriptive statistical data concerning publications, citations, co-citations, and the presence of keywords). Such analyses seem to be properly done and reasonable interpretation. The manuscript does not, however, include any astute inferential statistics or tests of significance, which could support statements of trend or gaps. Though not specifically required in bibliometrics, bibliometrics becomes more rigorous when confidence interval or statistical validation of trends is included or discussed. In the analyses reported, inferences can be drawn. The paper can be reinforced by the addition of further statistical evaluation, like to be done as the significance of trend or bibliometric network measures, consisting of more than counts and appearance representations. That would indicate strength of observed trends.
3. The author should consider adding a brief section to address some possible limitations of the study, including biases of the articles chosen or the literature gaps, which would enhance the conclusions and prove the critical view on the findings.

Is the work clearly and accurately presented and does it cite the current literature?

Partly

If applicable, is the statistical analysis and its interpretation appropriate?

Not applicable

Are all the source data underlying the results available to ensure full reproducibility?

Partly

Is the study design appropriate and is the work technically sound?

Yes

Are the conclusions drawn adequately supported by the results?

Yes

Are sufficient details of methods and analysis provided to allow replication by others?

Yes

Reviewer Expertise:

Business Analytics, Predictive analytics, Supply Chain, Artificial Intelligence, machine learning

I confirm that I have read this submission and believe that I have an appropriate level of expertise to confirm that it is of an acceptable scientific standard, however I have significant reservations, as outlined above.

## References

[1] : SPAR-4 and sustainable operations: A meta-analytic breakthrough. figshare. Dataset. *https://figshare.com/articles/dataset/SPAR-4_and_sustainable_operations_A_meta-analytic_breakthrough/28152632/1* .2025; 10.6084/m9.figshare.28152632.v1 10.6084/m9.figshare.28152632.v1 PMC1243895140963526

# Response

**Published:** 2025-08-13

**Authors:** vasim ahmad

Dear Editor

 Thank you for your useful comments and suggestions on the manuscript title “SPAR-4 and sustainable operations: A meta-analytic breakthrough" submitted in the F1000 Research Journal. We have modified the manuscript accordingly, and respective corrections are listed below:

**Reviewer Comments 1: **The article manuscript is structured and has a logical sequence, especially in the application of the SPAR-4 model. It has extensive coverage of recent and relevant literature, especially of the high-impact journals. However, clarity is compromised by more than a bit of jargon, repetition (in the introduction and methodology in particular), and a few grammatical inconsistencies. Other sources are obsolete, un-peer-reviewed datasets (e.g., Figshare) and can be low on academic rigor.

**Response: **Thanks for valuable suggestions. The modifications have been done and highlighted (yellow).

**Figshare:** We appreciate the concern regarding Figshare and similar repositories. In our manuscript, these sources are not used as primary evidence or theoretical input but solely to store and share supplementary materials (dataset, PRISMA checklist, and flow diagram). Figshare assigns Digital Object Identifiers (DOIs) and is widely recognised in the academic community as a credible platform for open data sharing. Its inclusion ensures transparency, reproducibility, and compliance with PRISMA guidelines, allowing other researchers to replicate or verify our results. We have clarified this distinction in the revised Methodology section.

**Reviewer Comments 2: **The manuscript article largely uses bibliometric analysis (descriptive statistical data concerning publications, citations, co-citations, and the presence of keywords). Such analyses seem to be properly done and reasonable interpretation. The manuscript does not, however, include any astute inferential statistics or tests of significance, which could support statements of trend or gaps. Though not specifically required in bibliometrics, bibliometrics becomes more rigorous when confidence interval or statistical validation of trends is included or discussed. In the analyses reported, inferences can be drawn. The paper can be reinforced by the addition of further statistical evaluation, like to be done as the significance of trend or bibliometric network measures, consisting of more than counts and appearance representations. That would indicate strength of observed trends.

**Response: **Thanks for valuable suggestions. Statistical validation has been included in the study (Section 4)

**Reviewer Comments 3: **The author should consider adding a brief section to address some possible limitations of the study, including biases of the articles chosen or the literature gaps, which would enhance the conclusions and prove the critical view on the findings.

**Response:**The modifications have been done and updated (Section 6)

 Sincerely

 Dr. Vasim Ahmad
